# Complete mitochondrial genome of *Micractinium pusillum* CCAP 231/1 (Chlorellaceae, Trebouxiophyceae)

**DOI:** 10.1080/23802359.2019.1698341

**Published:** 2019-12-11

**Authors:** Nam Seon Kang, Seung-Woo Jo, Jung A Lee, Kyeong Mi Kim, Hyeong Seok Jang, Eun Song Kim, Moongeun Yoon, Ji Won Hong, Ho-Sung Yoon

**Affiliations:** a Department of Taxonomy and Systematics, National Marine Biodiversity Institute of Korea, Seocheon, Republic of Korea;; b Department of Energy Science, Kyungpook National University, Daegu, Republic of Korea;; c School of Life Sciences, BK21 Plus KNU Creative BioResearch Group, Kyungpook National University, Daegu, Republic of Korea

**Keywords:** Complete mitochondrial genome, *Micractinium pusillum*, Chlorellaceae, Trebouxiophyceae

## Abstract

The mitochondrial genome of *Micractinium pusillum* CCAP 231/1 was completely sequenced. This mitogenome has 70,061 bp in length and consists of 62 genes including 32 protein-coding, 3 rRNA, and 27 tRNA genes. The overall GC content of the genome is 31.3%.

*Micractinium* Fresenius is a globally distributed genus in a variety of aquatic habitats. There are currently 16 taxonomically accepted species (Guiry and Guiry 2019). *Micractinium pusillum* Fresenius (Chlorellaceae, Trebouxiophyceae) is a holotype species of the genus and this species is known to be a cosmopolitan microalga commonly found in many types of freshwater environments (John et al. 2002). It is often considered as an indicator of nutrient-enriched water. In this study, the complete mitogenome of *M*. *pusillum* CCAP 231/1 was determined for the first time.

*Micractinium pusillum* CCAP 231/1 was purchased from the Culture Collection of Algae and Protozoa (CCAP). This strain was originally isolated from Wicken Lode, Cambridgeshire, England (52°18′ 24.29″N, 0°16′32.86″E). The culture was inoculated onto fresh BG-11 agar medium (UTEX, USA) containing imipenem (Sigma-Aldrich, St. Louis, USA) at a concentration of 100 µg ml^−1^ to suppress bacterial growth (Hong et al. 2015). A resulting single colony on the agar plate was then aseptically transferred to a liquid BG-11 medium and grown at 18 °C under cool fluorescent light (approximately 40 µmole m^−2^ s^−1^) in a light:dark cycle (14:10 hrs) for 4 weeks. Microalgal biomass was harvested by centrifugation at centrifugation at 2063 ×*g* (1580 R; Labogene, Daejeon, Korea). Whole genomic DNA was extracted from the sample using a DNeasy Plant Mini Kit (Qiagen, Hilden, Germany) followed by preparation of a library using an MGIEasy DNA Library Prep Kit V1 (BGI, Shenzhen, China) according to the manufacturer’s instruction. Whole genome sequencing was performed using BGISEQ-500 (BGI, China) sequencer and raw data were filtered to obtain >10 Gb clean data per each sample. *De novo* mitogenome assembly was carried out using NOVOPlasty v3.6 software (Dierckxsens et al. 2017). The size of the circular mitogenome produced is 70,061 bp (GenBank accession number MN649871) which is smaller than that of the previously reported *M*. *conductrix* mitogenomes (74,708 bp, GenBank accession number KY629619). The nucleotide composition is 34.6% A, 34.1% T, 15.2% G, and 16.0% C. The overall GC content is 31.3%. The *M*. *pusillum* mitogenome contains 62 genes, including 32 predicted protein-coding, 3 rRNA, and 27 tRNA genes. Among 32 genes, 19 genes were revealed as complete protein-coding genes, which all of them were started with ATG as a start codon and ended by TAA as a stop codon. It was found that there was only 1 case of gene-overlapping with a size of 85 bp and 27 tRNA genes ranged from 31 to 86 bp in length.

Phylogenetic analysis was carried out by PhyML 3.0 with 11 reported mitogenome sequences (Fan et al. 2017) belonging to the Trebouxiophyceae family and it was visualized by FigTree v1.4.4. The result showed the phylogenetic position of *M*. *pusillum* CCAP 231/1 (Figure 1) within this family. This new information would contribute to a better understanding of the phylogenetic relationships of the *Micractinium* species and mitochondrial genome diversity and evolution in the Trebouxiophyceae.

**Figure 1.**
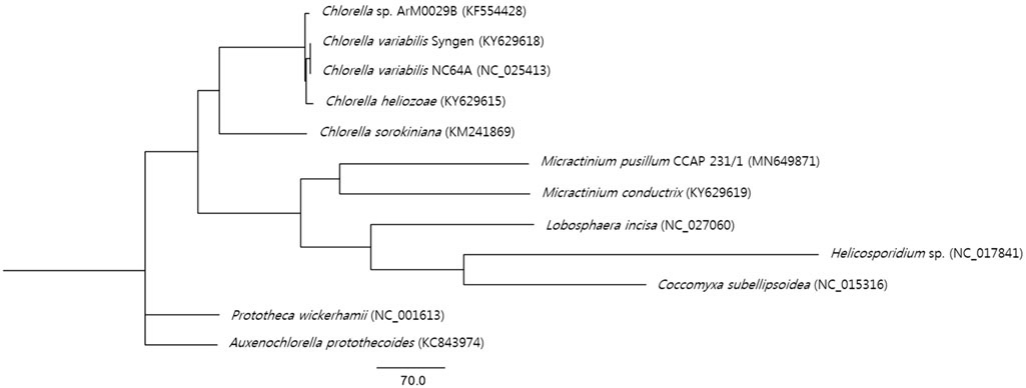
Maximum-likelihood phylogenetic tree of *M. pusillum* CCAP 231/1 and 11 other species. GenBank accession numbers were indicated in the parentheses.
